# Zscan4 as a Candidate Conveyor of Early Developmental Defects in O-GlcNAc Transferase Intellectual Disability

**DOI:** 10.1016/j.mcpro.2025.101077

**Published:** 2025-09-29

**Authors:** Veronica M. Pravata, Hao Jiang, Andrew T. Ferenbach, Angus Lamond, Daan M.F. van Aalten

**Affiliations:** 1 Molecular, Cell and Developmental Biology Division, School of Life Science, University of Dundee, Dundee, UK; 2 Department of Molecular Biology and Genetics, Aarhus University, Aarhus, Denmark

**Keywords:** OGT-CDG, O-GlcNAcylation, O-GlcNAc transferase, TET, neurodevelopment, pluripotency

## Abstract

Variants in the human β-*N*-acetylglucosamine (O-GlcNAc) transferase (*OGT*) gene give rise to an intellectual disability (ID) syndrome termed OGT congenital disorder of glycosylation (OGT-CDG). The mechanisms by which loss of OGT and/or protein O-GlcNAcylation leads to this syndrome are not understood, but symptoms associated with the syndrome suggest a developmental origin. Here, we establish and characterize two lines of mouse embryonic stem cells carrying different patient mutations and show that these mutations lead to disrupted O-GlcNAc homeostasis. Using quantitative proteomics on these cells in the pluripotent state, we identify candidate proteins/pathways that could underpin this syndrome. In addition to the increased levels of OGT and decreased levels of OGA reflecting disrupted O-GlcNAc homeostasis, we find that expression of the ID gene *Zscan4* is upregulated. This is associated with increased levels of the OGT:10 Eleven (Tet) - protein complex that regulates DNA methylation and *Zscan4* expression. These data uncover a potential mechanism contributing to the developmental aspects of OGT-CDG.

## Highlights

• Proteomics pinpointed protein candidates that may underlie OGT-CDG.
• Zscan4 is upregulated and linked to higher OGT:Tet Eleven protein complex.
• Early O-GlcNAcylation defects may cause transpcriptional and epigenetic dusregulation.

## Graphical Abstract

**Figure:**
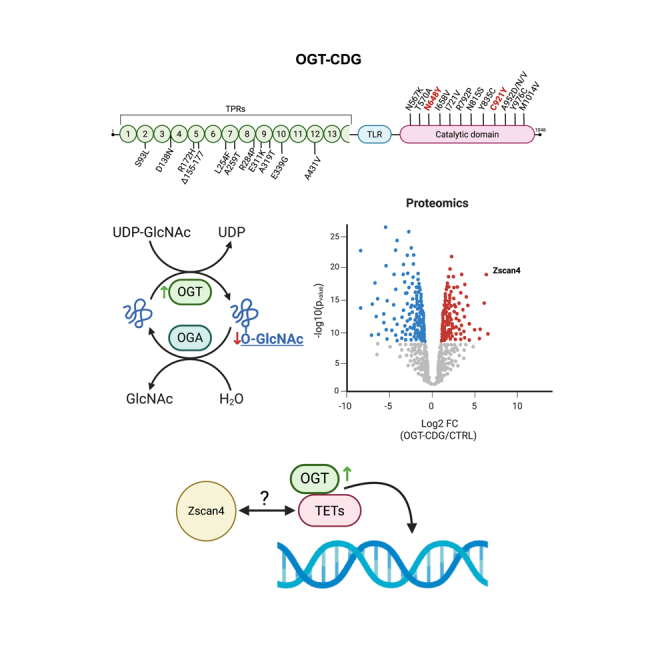

## In Brief

Mutations in the O-GlcNAc transferase (*OGT*) gene cause OGT-CDG, a neurodevelopmental disorder marked by intellectual disability and developmental delay. Using quantitative proteomics, this study identifies deregulated proteins in OGT-CDG, including upregulation of Zscan4 linked to increased OGT:Tet protein complex levels. These findings suggest that impaired O-GlcNAcylation during early development disrupts transcriptional and epigenetic regulation, offering new insight into the molecular mechanisms of OGT-CDG and advancing our understanding of glycosylation-related neurodevelopmental disorders.

Protein O-GlcNAcylation is a dynamic posttranslational modification that involves the transfer of a single O-GlcNAc moiety onto serine/threonine residues of nucleocytoplasmic proteins (1). This is mediated by the glycosyltransferase OGT, which utilizes UDP-GlcNAc as substrate, the final product of the hexosamine biosynthetic pathway (HBP) (2). Hydrolysis of O-GlcNAc from the modified proteins is mediated by the O-GlcNAcase (OGA) (3). O-GlcNAcylated proteins are known to modify more than 6000 proteins in *Homo sapiens* (4). These proteins include kinases (5), mitochondrial proteins (6, 7), structural proteins (8), transcription and epigenetic factors (9), nuclear porins (10), and components of vesicular trafficking pathways (11). O-GlcNAcylation of proteins can have different effects, spanning from alteration in enzymatic activity, protein stability, subcellular localization, and interaction with binding partners (12, 13, 14). In addition to its glycosyltransferase catalytic function, OGT plays an important role in several aspects of cellular physiology, such as the cleavage of the Host Cell Factor 1 (HCF1) (15, 16), which is crucial for cell cycle progression from the S to G2/M phase and transcription (17, 18, 19). Additionally, OGT interacts with several factors involved in chromatin remodeling, such as Ten-11 translocation 1 to 3 (TET1-3) proteins (20, 21), Sin3 (22) and HDACs (23, 24) modulating activation, or repression, of specific subsets of regulatory genes.

The essential role of O-GlcNAc in development has been demonstrated by a range of genetic studies where deletion of *OGT* resulted in embryonic lethality (25) Jan 9). In *Drosophila melanogaster*, abrogation of the Polycomb group gene super sex combs (*sxc*), which codes for OGT, results in pupal lethality associated with homeotic transformations (26, 27, 28, 29). Similarly, knockout of *Ogt* in *Mus musculus* and mouse embryonic stem cells (mESCs) resulted in cell death (30, 31). O-GlcNAcylation is thought to play a critical role in embryonic development, where it is at its highest levels (32), and in the modulation of cellular fate (33, 34, 35). Post-partum, total O-GlcNAc levels plateau and are then stable for most adulthood (36). O-GlcNAc is particularly abundant in brain, in particular in specific brain regions in both rodents and humans, such as the hippocampus, and in certain cellular subtypes such as CA1 pyramidal cells, Purkinje cells, GABAergic interneurons, and astrocytes (37, 38, 39, 40). Mass spectrometry analyses have identified several neuronal specific O-GlcNAc proteins, such as the transcription factor cAMP responsive element-binding protein (CREB) (41, 42), the Collapsin Response Mediator Protein-2 (CRMP2) (43), and the Ca^2+^/calmodulin-dependent protein kinase (CAMKII) (44). Additionally, O-GlcNAc is particularly enriched in synapses and synaptic vesicles, with more than 20% of all synaptic proteins being modified (45, 46, 47). Fluctuations in the physiological levels of protein O-GlcNAcylation have been associated with neurodegeneration, such as Alzheimer’s and Parkinson’s disease (13, 48, 49).

**Fig. 1:**
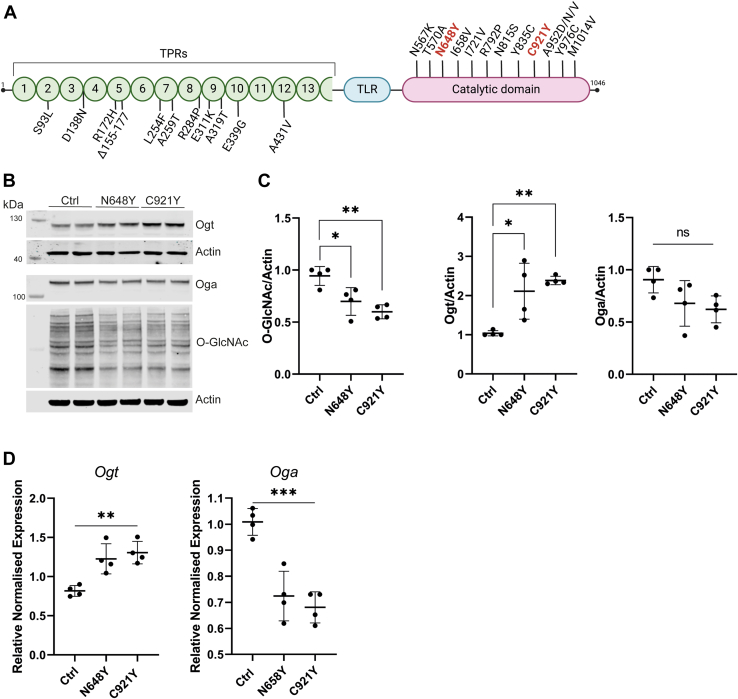
**OGT-CDG mutations lead to altered O-GlcNAc homeostasis in naive conditions.***A*, schematic representation of the OGT structure. The OGT protein comprises an N-terminal Tetratricopeptide Repeat (TPR) domain (*green*), a tetratricopeptide repeat-like (TLR) domain in cyan, and a C-terminal catalytic domain (*magenta*). Highlighted along the structure are specific mutations associated with OGT-CDG, with the bolded mutations representing those investigated in this study. *B*, immunoblots on full cell lysate of Control (Ctrl), N648Y and C921Y mESCs showing Ogt, Oga, O-GlcNAc and Actin protein levels. *C*, quantification of Western blotting of Ogt, Oga and O-GlcNAc levels normalizsed to actin signal. n = 4, mean ± SD. One-way ANOVA. ∗ <0.05, ∗∗ <0.005. *D*, qPCR analysis of gene expression of *Ogt* and *Oga* in undifferentiated mESCs. N = 4, mean ± SD. One-way ANOVA, ∗∗<0.005, ∗∗∗<0.0005.

Recently, variants in *OGT* have been associated with a new syndromic form of intellectual disability, termed OGT Congenital Disorder of Glycosylation (OGT-CDG) (50, 51, 52, 53, 54, 55, 56, 57). Missense variants in patients with OGT-CDG are located in both the tetratricopeptide (TPR) and catalytic domains of OGT (Fig. 1*A*). All patients display developmental delay, an IQ < 80 and a degree of facial dysmorphia (51, 58), suggesting that different mutations in *OGT* could have a similar etiology. However, our understanding of the mechanistic biology underpinning OGT-CDG remains limited. The early-onset symptoms, including global developmental delay and facial dysmorphisms evident at birth, suggest disrupted developmental processes, perhaps in addition to neurophysiological alterations, such as changes in synaptic activity, signal transmission, or neuronal plasticity, that typically manifest later in life.

Here, we used 2 mouse embryonic stem cell (mESC) lines carrying different OGT-CDG mutations as a model system to enable the investigation of how OGT variants disrupt fundamental early developmental pathways. We show that in their naïve state, these OGT-CDG lines display disrupted O-GlcNAc homeostasis, with reduced levels of O-GlcNAcylation despite increased levels of Ogt at the protein level. By using isobaric labeling tandem mass tags (TMT) proteomics of these mESCs in their pluripotent state, we identify upregulation of Zscan4, a protein critical to the successful progression of early embryonic development and totipotency. We demonstrate that this may occur due to the interaction between Ogt and the TET family of proteins, which are known to regulate transcription through epigenetic modifications contributing to the altered transcriptional landscape observed in OGT-CDG mESCs. These findings highlight how defects in O-GlcNAcylation early in development may lead to transcriptional and epigenetic dysregulation that could contribute to symptoms associated with OGT-CDG.

## Experimental Procedures

### Mouse ES Cell Culture

The mESC E14-TG2a.IV AW2 line was acquired from the MRC Centre for Regenerative Medicine, Institute for Stem Cell Research, University of Edinburgh. Generation of N648Y and C921Y mESCs has been described elsewhere (52, 53). mESCs were cultured in a naive undifferentiated state according to Smith’s lab guidelines for no longer than 30 passages (59). Briefly, cells were kept in 0.2% gelatin [w/v] coated plates in 2i/LIF medium, which contains DMEM/F12 (Gibco) and Neurobasal (Gibco) in a 1:1 ratio, B27 (1X, Invitrogen 17504044), N2 supplement (1X, made in house), 50 μM β-mercaptoethanol (Thermo Fisher 31350010), 2 mM L-glutamine (Thermo Fisher - 25030081), 0.1% sodium bicarbonate (Thermo Fisher - 25080094), 0.11% bovine serum albumin fraction V (Thermo Fisher - 15260037), 12.5 μg/ml human recombinant insulin (Thermo Fisher - 12585014), 1 μM PD0325901 (Sigma - P2499), 3 μM CHIR99021 (Sigma - SML1046) and 100 units/ml LIF (produced in house) at 37 °C in 5% CO_2_. Cells were passaged every 2 days, using Accutase (Sigma - A6964) for detaching them, and cells were counted with a Luna cell counter and plated at the appropriate density according to Mulas and colleagues.

### Experimental Design and Statistical Rationale

Three independent biological replicates were analyzed per condition: wild-type control, C921Y, and N648Y OGT-CDG mESC lines. For each replicate, cells were independently cultured, lysed, digested, and labeled using the TMT 10plex system. Labeled peptides were pooled and fractionated by high-pH reversed-phase chromatography before LC-MS/MS. The experiment was designed to minimize technical variation by including all samples in a single multiplexed TMT experiment. Quantitative analysis was performed using MaxQuant (v1.6.7.0), and protein-level expression changes were assessed by two-sided unpaired *t* tests. Proteins with a log_2_ fold change greater than ±0.2 and *p* < 0.05 were considered significantly deregulated. This combination of biological replication and isobaric labeling enables statistically robust detection of proteome-wide alterations associated with OGT-CDG mutations.

### Tandem Mass Tag Sample Preparation

1.12 × 10^6^ cells were plated in T-75 flasks at cell passage 16. After 48 h, cells were resuspended using Accutase at room temperature for 6 min and washed twice with PBS. Cells were resuspended in 1.5 ml lysis buffer (8 M urea in 50 mM triethyl ammonium bicarbonate (TEAB)) and mixed at room temperature for 15 min. Cellular DNA was sheared using Pierce Universal Nuclease for Cell Lysis (Thermo Fisher – 88702) for 10 min at 37 °C and ultrasonicating the lysates at 4 °C for 10 cycles of 30 s. Proteins were reduced using tris-carboxyethyl phosphine (TCEP, 25 mM) for 30 min at room temperature, then alkylated in the dark for 45 min using iodoacetamide (50 mM). Total proteins were quantified using Pierce 660 nM Protein Assay Kit (Thermo Fisher – 22662), and 200 μg of protein was prepared for each sample. Lysates were diluted 4-fold (∼2 M urea concentration) and Lys-C was added at a 1:100 ratio for 4 h at 37 °C. Samples were further diluted 2.5 times (∼0.8 M final concentration of urea), and trypsin was added to a 1:50 ratio overnight at 37 °C. Reactions were stopped by acidification with trifluoroacetic acid (TFA) to a final concentration of 1% (v:v). Peptides were desalted using C18 Sep-Pak cartridges (Waters) following the manufacturer’s instructions and dried in vacuo. Peptides were resuspended in 150 μl of 100 mM TEAB and quantified using Pierce Quantitative Colorimetric Peptide Assay (Thermo Fisher – 23275). Peptides from each sample were labeled with TMT10plex Isobaric Label Reagents by following its user guides (Thermo Fisher - 90110) (60). For tandem mass tag (TMT)-based MS quantification, 5 μg of labeled samples were taken to check incorporation efficiency, while 100 μg of labeled peptides from each sample were mixed in equal amounts and dried in vacuo. The TMT-labeled samples were fractionated using off-line high-pH reverse-phase (RP) chromatography following Bernes and colleagues' guidelines (61); Briefly, samples were loaded onto a 4.6 × 250 mm Xbridge BEH130 C18 column with 3.5 μm particles (Waters). Using a Dionex bioRS system, the samples were separated using a 25-min multistep gradient of solvents A (10 mM formate at pH 9) and B (10 mM ammonium formate, pH 9 in 80% acetonitrile), at a flow rate of 1 ml min−1. Peptides were separated into 48 fractions, which were consolidated into 24 fractions. The fractions were subsequently dried, and the peptides dissolved in 5% formic acid and analyzed by LC–MS/MS.

Samples were analyzed using a Q Exactive Plus Hybrid Quadrupole-Orbitrap Mass Spectrometer (Thermo Scientific), equipped with a Dionex ultra-high-pressure liquid-chromatography system (RSLCnano). RPLC was performed using a Dionex RSLCnano HPLC (Thermo Scientific). Peptides were injected onto a 75 μm × 2 cm PepMap-C18 pre-column and resolved on a 75 μm × 50 cm RP-C18 EASY-Spray temperature-controlled integrated column-emitter (Thermo Scientific), using a 100 min multistep gradient from 10% B to 32% B with a constant flow of 270 nl min^−1^. The mobile phases were: 2% ACN incorporating 0.1% FA (solvent A) and 80% CAN incorporating 0.1% FA (solvent B). The spray was initiated by applying 2.5 kV to the EASY-Spray emitter.

The data were acquired under the control of Xcalibur software in a data-dependent acquisition mode (DDA). The survey scan was acquired in the Orbitrap covering the m/z range from 375 to 1600 Da at a mass resolution of 70,000 with an automatic gain control (AGC) target of 3.0 × 10^6^ ions and 50 ms Maximum IT. MS/MS was performed in the Orbitrap at a mass resolution of 35,000, with an AGC target of 2.0 × 10^5^ and 120 ms Maximum IT. The fragmentation of the top 14 intensive ions from MS1 was performed using an HCD collision energy of 35% with an isolation window of 0.7 m/z. The mass spectrometry proteomics data have been deposited to the ProteomeXchange Consortium via the PRIDE partner repository with the dataset identifier PXD064425 (Token: xYrFBA5sDqth).

### Identification and Quantification

For TMT-MS data analysis, the data from all high-pH RP fractions were analyzed together using MaxQuant (v1.6.7.0) with default settings unless otherwise specified. Peptide and protein identifications were filtered at a 1% false discovery rate (FDR) at both the Peptide Spectrum Match (PSM) and protein levels. The search was performed against the *M. musculus* UniProtKB/Swiss-Prot reference proteome (Proteome ID: UP000000589, downloaded in April 2020), containing 17,108 reviewed protein entries. The database was searched using a precursor ion mass tolerance of 4.5 ppm and a fragment ion mass tolerance of 20 ppm. The quantification type was set to Reporter ion MS2 with TMT 10plex. Carbamidomethylation of cysteine was set as a fixed modification, while methionine oxidation and protein N-terminal acetylation were set as variable modifications. Trypsin/P was specified as the digestion enzyme, allowing for a maximum of two missed cleavages. Data used for downstream analysis were obtained from the proteinGroups.txt output file. Reverse hits, contaminants, and proteins identified only by site were excluded from further analysis.

Data analysis was conducted using R, primarily leveraging the DEP and dplyr packages. Raw protein data were filtered to remove reverse sequences, potential contaminants, and proteins only identified by site. Unique protein identifiers were generated using gene names and protein IDs for downstream analysis. To handle missing values, proteins identified in at least two of three replicates for each condition were retained, and missing values were imputed using a k-nearest neighbor (KNN) approach. The filtered and imputed data were normalized using variance stabilizing normalization (VSN), and various data visualizations were created using ggplot2 and DEP to assess data quality.

For differential enrichment analysis, a linear model with empirical Bayes statistics was applied, testing the OGT-CDG mutant samples (C921Y and N648Y) against a common wild-type control. Significant proteins were identified based on a *p*-value cutoff of <0.05 and a log2 fold change cutoff of 1.5.

Further analysis was performed to identify proteins commonly deregulated in both OGT-CDG genotypes using Venn diagrams generated with the VennDiagram package (62). Correlation plots were produced to assess the relationship between the log2 fold changes of C921Y and N648Y versus the control. Gene ontology (GO) and pathway enrichment analyses were conducted on the common deregulated proteins using gProfiler to identify relevant biological pathways and processes. The DisGeNET package (63) was used to retrieve gene-disease associations linked to neurodevelopmental disorders. Genes related to intellectual disability and microcephaly were identified from a curated disease database and were cross-referenced with the proteomics data to identify deregulated proteins associated with these conditions. Only genes associated with the selected neurodevelopmental and cortical malformation UMLS codes in the DisGeNET CURATED database are included.

### Western Blotting

Cells were lysed in RIPA buffer (150 mM NaCl, 1% Nondiet P-40, 0.5% Sodium deoxycholate, 0.1% SDS, 25 mM Tris HCl pH 7.5, 1 mM Na3VO4, 50 mM NaF and 5 mM Na4P2O7) with freshly added Protease Inhibitor Cocktail, vortexed and kept on ice for 30 min. Lysates were spun down at max speed for 20 min, and supernatants were then aliquoted into a fresh Eppendorf. Total protein was quantified using Pierce 660 nM Protein Assay Kit (Thermo Fisher – 22662), and 20 μg of protein was resuspended in NuPAGE LDS Sample buffer (Thermo Fisher – NP0007) in a final dilution of 1X. Samples were then heated at 70 °C for 10 min and loaded onto a NuPAGE 4 to 12%, Bis-Tris, 1.0 mm, Mini Protein Gel (Thermo Fisher - NP0321) in 1X NuPAGE MOPS SDS Running Buffer (Thermo Fisher – NP0001) and run for approximately 2 h at 100 V. Gels were then transferred into 0.45 μM nitrocellulose membrane (GE Healthcare – 10600002) using Mini Trans-Blot Electrophoretic Transfer Cell tanks (Bio-Rad) in Towbin buffer (25 mM Tris, 192 mM glycine pH 8.3, 20% methanol) for 1 h at 100 V. Membranes were then blocked in 5% [w/v] BSA Fraction V (Roche – 107350940) in PBS-T (Tween 0.01% [v/v]) for 1 h at room temperature and primary antibodies were incubated overnight at 4 °C (see Supplemental Table S2). Membranes were washed three times with PBS-T for 10 min each and incubated in secondary antibody for 45 min at room temperature in the dark. Membranes were finally washed three times in PBS for 10 min and detected using the LI-COR Odyssey Scanner. Images were then analyzed using Image Studio Lite Version 5.2 and statistical analyses were carried out using GraphPad Prism 10.

### Real-Time PCR Analysis from mESCs

Cells were cultured in 2i/LIF media as described above and plated in 6-well plate wells at a density of 1.4 × 105 cells/well. Cells were detached using Accutase, and total RNA extraction was performed using GeneJet RNA extraction Kit (Thermo). 500 ng of sample RNA was subjected to reverse transcription using qScript cDNA Synthesis Kit (Quantabio) according to the manufacturer’s guidelines. qPCR was performed using Luna Universal qPCR Master Mix (NEB). All qPCR analyses were performed at 95 °C for 30 s and then 40 cycles of 95 °C for 5 s, 60 °C for 15 s and 68 °C for 10 s. The threshold crossing value was noted for each transcript and normalized to the internal controls 18S and Actb. The relative quantification of each mRNA was performed using the comparative Ct method. Experiments were performed using CFX Connect Real-Time PCR Detection System (BioRad), and data processing was performed using CFX Manager Softer (BioRad). Samples were assayed in triplicate using the thermocycler profile conditions described above. Standard curves were assayed for each primer. Primers are listed in the Appendix, Supplemental Table S1.

### Immunoprecipitation of Nuclear Extracts

Cells were cultured in 2i/LIF media, passage 16 and 17 were used, with two different clones for each passage. 2 T-75 plates were used for each biological replicate, and cells were seeded at a density of 1.1 × 10^6^ cells/well. After 48 h, cells were detached using Accutase (∼2 ml per T75), and cells were resuspended in 10 ml DPBS. Nuclear enrichment is adapted from 495. Briefly, cell pellets were re-suspended in 5 ml of ice-cold Buffer A (10 mM Hepes, pH 7.9, 1.5 mM MgCl_2_, 10 mM KCl, 0.5 mM DTT, Protease Inhibitor Cocktail, 1 μM GlcNAcstatin G) and incubated on ice for 5 min. The re-suspended pellet was then transferred into a pre-chilled 7 ml Dounce homogenizer, and cells were broken open using 10 strokes of a tight pestle. Cells were centrifuged for 5 min at 4 °C, 1000 rpm. The nuclear pellet was then resuspended in 3 ml of S1 (0.25 M Sucrose, 10 mM MgCl_2_) and layered over a 3 ml cushion of S2 (0.35 M Sucrose, 0.5 mM MgCl_2_) by slowly pipetting S1 solution on top of S2. The gradient was centrifuged for 10 min at 4 °C, 3500 rpm and the pellet was retained as the nuclear fraction. Nuclear pellet was resuspended in 400 μl of High Salt Buffer (300 mM NaCl, 20 mM Tris-HCl, pH 7.5, 0.2% NP40, 10% glycerol, 1 μM GlcNAcstatin G, 1x Halt Phosphatase Inhibitor Cocktail, Protease Inhibitor Cocktail) and left on ice for 15 min. Lysate was passed through a 21 g needle 5 times and subsequently centrifuged for 10 min at 4 °C, 3500 rpm. Supernatant was transferred into a new tube and quantified using Pierce 660 nM. 100 μg of nuclear extract was then aliquoted into a new tube and resuspended in a final volume of 300 μl. Either TET2 antibody (D6C7K, CST, 1:75 [vol/vol]) or OGT antibody (ab96718, Abcam, 1 μg for each 100 μg of lysates) was used for immunoprecipitation, and 2 μg of Rabbit IgG (2729, CST) was used as a negative control. Lysates were incubated at 4 °C for 2 h on a rolling platform, and then incubated with 25 μl of Dynabeads protein G magnetic beads for each sample and incubated for further 2 h at 4 °C on a rolling platform. Beads were washed twice with 300 μl of High-Salt Buffer. For the third wash, the beads were resuspended in 150 μl of High Salt Buffer and transferred into a clean tube to avoid co-elution of proteins bound to the tube wall. The IPed proteins were eluted using 20 μl of 50 mM glycine (pH 2.7) and 10 μl of 4x LDS. Samples, which included 10 μg of Inputs, were boiled for 10 min at 70 °C and loaded into a 4 to 8% Tris-acetate gels. Gels were transferred overnight (16 h) using a Nitrocellulose membrane (0.2 μM) at 10V (Used Biorad Trans-Blot Cell). Membranes were blocked for 1 h in 5% BSA in TBS-T (0.1% Tween) and incubated at 4 °C overnight with antibodies (see Supplemental Table S2). The following day, membranes were washed in TBS-T and incubated for 45 min with secondary Ab (Rabbit 800 nM and Mouse 680 nM for the Licor System). Membranes were washed and detected using the Licor System.

## Results

### Naive OGT-CDG mESCs Show Disrupted O-GlcNAc Homeostasis

To elucidate the potential mechanisms underlying developmental delay and defects in OGT-CDG, we focused on two recently identified mutations near the active site, N648Y (53) and C921Y (52) that are both associated with facial dysmorphia and intellectual disability (Fig. 1*A*). These variants were previously generated using CRISPR/Cas9-mediated gene editing at the endogenous locus in male mouse embryonic stem cells (mESCs), weakly maintaining pluripotency through Leukemia Inhibitory Factor (LIF) only. For this work, three independent clones were generated for each variant and cultured in 2i+LIF media containing a GSK3 inhibitor (CHIR99021) and a MEK inhibitor (PD0325901) in addition to LIF to generate a more homogeneous population in a naïve, ground state of pluripotency resembling the pre-implantation embryonic stage (64). To understand whether these different variants had similar effects on O-GlcNAc homeostasis, we assessed the levels of protein O-GlcNAcylation using western blotting (Fig. 1, *B* and *C*). As previously reported for these variants, the O-GlcNAc levels in both OGT-CDG mESCs appear to be reduced compared to control cells. The levels of Ogt were higher in both types of OGT-CDG mESCs (n = 4, mean ± SD, one-way ANOVA, ∗ corresponds to *p* < 0.05 and ∗∗ to *p* < 0.005*)*, while average Oga levels were lower but not significantly so. *Ogt* mRNA levels were also significantly increased in OGT-CDG mutations compared to control cells (mean ± SD, n = 4. ∗∗ corresponds to *p* < 0.005), while *Oga* mRNA levels were significantly reduced (mean ± SD, n = 4. ∗∗∗*p* < 0.0005, Fig. 1*D*). These findings are in line with the previously proposed notion that feedback mechanisms between O-GlcNAc, Ogt and Oga maintain O-GlcNAc homeostasis (51, 52, 53, 54, 57). Taken together, these data show that naïve OGT-CDG mESCs show disrupted O-GlcNAc homeostasis.

### OGT-CDG Mutations Lead to Changes in ID/DD Proteins in the mESC Proteome

**Fig. 2:**
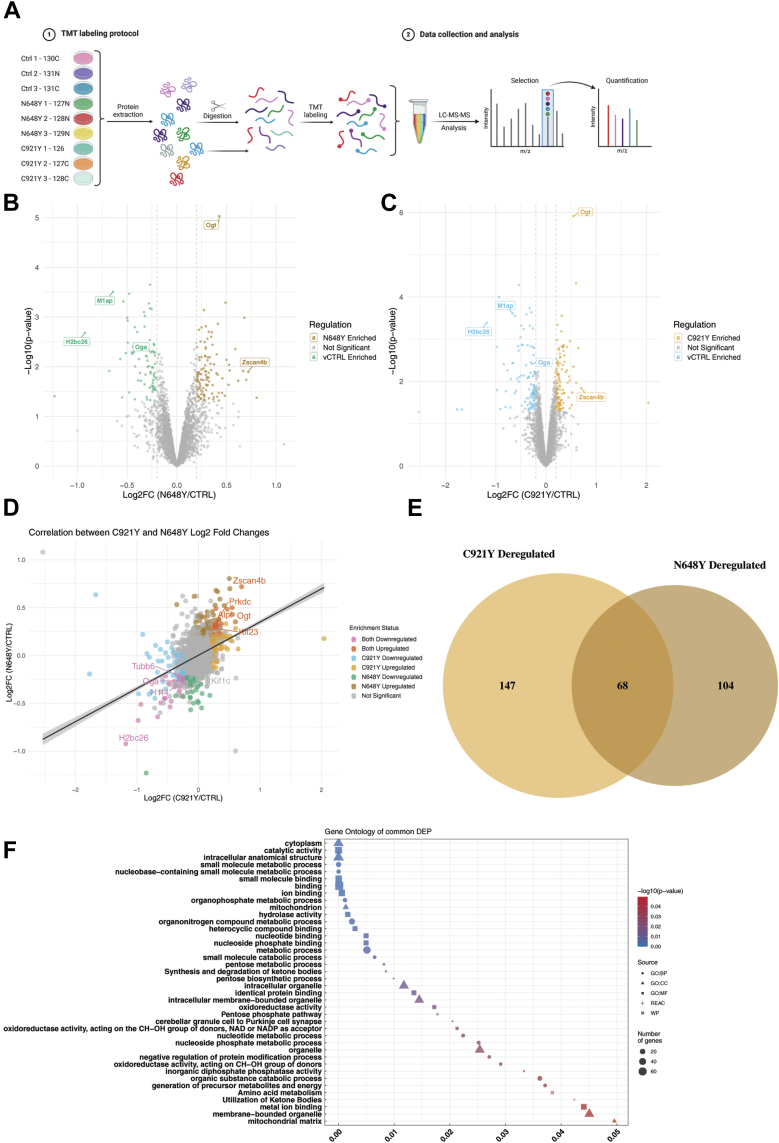
**Tandem Mass Tag (TMT) proteomics reveals changes in OGT-CDG mESCs.***A*, schematic representation of the TMT workflow used for proteomics analysis. The workflow includes protein extraction, digestion, TMT labeling of samples, data collection, and analysis. Sample labels include controls and OGT-CDG mutants (N648Y and C921Y), with corresponding isobaric tags shown. Created in https://BioRender.com. *B*, Volcano plot illustrating the enrichment and regulation status of differentially expressed proteins (DEPs) in N648Y OGT-CDG mutant compared to control. The log_2_ fold changes are plotted against −log_10_(*p*-value), highlighting upregulated (N648Y Enriched), downregulated (CTRL Enriched), and non-significant DEPs. Significant proteins, including Btrc, M1ap, Ogt, and Zscan4, are labeled. *C*, Volcano plot illustrating the enrichment and regulation status of DEPs in C921Y OGT-CDG mutant compared to control. The log_2_ fold changes are plotted against −log_10_(*p*-value), highlighting upregulated (C921Y Enriched), downregulated (CTRL Enriched), and non-significant DEPs. Significant proteins, including Btrc, M1ap, Ogt, and Zscan4, are labeled. *D*, scatterplot of log2 fold changes of proteins deregulated in C921Y versus N648Y mESCs compared to wild type (WT) mESCs (Pearson’s correlation coefficient = 0.53). Each dot represents a protein. Proteins that meet the significance threshold for upregulation in both C921Y and N648Y mESCs are coloured in soft red, while proteins that are significantly downregulated in both genotypes are coloured in soft *purple*. Proteins uniquely upregulated or downregulated in C921Y are shown in muted *orange* and muted *blue*, respectively, while those uniquely upregulated or downregulated in N648Y are displayed in *light brown* and muted *green*, respectively. Non-significant hits are shown in *light grey*. A linear regression line (*black*) illustrates the overall trend between the two genotypes. *E*, Venn diagram showing the overlap of deregulated proteins in C921Y and N648Y OGT-CDG mESCs compared to control mESCs. Deregulated proteins were defined as those with a log2 fold change >0.2 or < −0.2 and a *p* < 0.05. The orange circle represents proteins deregulated in C921Y, while the *brown* circle represents those deregulated in N648Y. The intersection highlights the proteins that are commonly deregulated in both genotypes. *F*, Gene Ontology (GO) and pathway enrichment analysis of proteins commonly deregulated in C921Y and N648Y OGT-CDG mESCs, based on GO, Reactome, KEGG, and WikiPathways databases. The x-axis lists the enriched terms, ranked by *p* value, with -log10(*p*) indicated by the color gradient from *blue* (higher *p* values) to *red* (lower *p* values). The size of the points corresponds to the number of genes overlapping with each term, while the shape of the points indicates the source database. Terms related to biological processes, molecular functions, and pathways involved in neurodevelopment and cellular processes are highlighted.

To gain deeper insights into the molecular consequences of OGT-CDG variants in naïve mESCs, we performed TMT-based proteomics to examine proteome-wide changes (Fig. 2*A*). Proteins were extracted using lysis buffer, followed by reduction, alkylation, and tryptic digestion. The resulting peptides from different samples were labeled with a TMT 11-plex kit, pooled, and processed through high-pH fractionation, then analyzed via high-resolution liquid chromatography coupled with tandem mass spectrometry (LC-MS/MS). This analysis identified 4192 proteins with high confidence. Each OGT-CDG line (C921Y and N648Y) was analyzed individually against the control to identify proteome-wide changes specific to each variant. Proteins were considered as hits if their levels in OGT-CDG vs. control mESCs showed log2 ratio (OGT-CDG/Control) < −0.2 (0.87-fold change) or > 0.2 (1.15-fold change) with *p* < 0.05 (Fig. 2, *B* and *C*). Given the subtle nature of proteome alterations expected from point mutations in Ogt, the ±0.2 log_2_FC cutoff was chosen to capture even modest but biologically meaningful changes in protein abundance. Next, we compared these proteomic changes across the two independent OGT-CDG mESC lines, relative to an isogenic wild-type control, to assess the similarity of proteome alterations between these genotypes (Fig. 2*D*). Pearson correlation analysis of log2 fold change values for all detected proteins in the two OGT-CDG lines revealed a moderate positive correlation (r = 0.52). This indicates that, although both genotypes produce globally similar changes in the mESC proteome, there are some differences in the direction and magnitude of protein alterations between them, perhaps reflecting the heterogeneity and incomplete penetrance of symptoms associated with OGT-CDG (51, 53). These differences likely reflect underlying molecular divergences in proteome regulation and O-GlcNAcylation dynamics in each genotype. We focused on identifying proteins that changed similarly in both genotypes. From our analysis, we identified a subset of 68 proteins that were either upregulated or downregulated in both C921Y and N648Y (Fig. 2, *D* and *E*). Gene Ontology (GO) analysis of these proteins revealed their involvement in key biological processes such as intracellular anatomical structure, mitochondrial function, and the pentose phosphate pathway (Fig. 2*F*). Among the deregulated proteins, we observed consistent upregulation of Ogt in both C921Y and N648Y genotypes, aligning with the disrupted O-GlcNAc homeostasis described earlier. Interestingly, while significant Oga downregulation was previously detected only at the mRNA level, this proteomic analysis also suggests only subtle changes at the protein level. The incongruence between protein and mRNA level changes may reflect the different sensitivities of the methods used, but together further support the notion of O-GlcNAc dyshomeostasis in OGT-CDG. We next focused on proteins previously linked to intellectual disability (ID) and developmental disorders (DD), identifying several candidates associated with microcephaly or intellectual disability using DisGeNET, a curated database of gene-disease associations (Table 1). These proteins, affected by both OGT-CDG variants, suggest potential disruptions in neurogenesis and neurodevelopmental processes. Given the neurological features observed in OGT-CDG patients, these candidates warrant further investigation to understand how their dysregulation could contribute to the pathogenesis of ID/DD. For example, Meiosis 1 arrest protein (M1ap) is involved in developmental signaling pathways crucial for cell cycle regulation, and its deregulation affects cellular division and growth (65, 66). Zinc finger and SCAN domain-containing protein 4 (Zscan4), a key player in chromatin remodeling and pluripotency (67), was upregulated in both OGT-CDG variant mESC lines, indicating potential disruptions in chromatin structure and gene expression that could further influence cellular differentiation and development. In summary, our findings reveal a complex landscape of proteomic alterations in OGT-CDG mESCs. Dysregulated proteins point to disruptions in neurodevelopmental pathways, chromatin remodeling, and cellular metabolism, linked to ID/DD syndromes.

**Table 1:** Deregulated Proteins linked to Neurodevelopmental Disorders according to DisgeNet

gene_symbol	disease_name	Score	Description
GDI1	Mental retardation, nonspecific	0.8	GDP dissociation inhibitor 1 [Source:HGNC Symbol;Acc:HGNC:4226]
H1-4	Neurodevelopmental Disorder	0.65	H1.4 linker histone, cluster member [Source:HGNC Symbol;Acc:HGNC:4718]
PRKDC	microcephaly (physical finding)	0.45	protein kinase, DNA-activated, catalytic subunit [Source:HGNC Symbol;Acc:HGNC:9413]
KIF23	microcephaly (physical finding)	0.4	kinesin family member 23 [Source:HGNC Symbol;Acc:HGNC:6392]
FCSK	Neurodevelopmental Disorder	0.4	fucose kinase [Source:HGNC Symbol;Acc:HGNC:29500]
ALPL	microcephaly (physical finding)	0.4	alkaline phosphatase, biomineralization associated [Source:HGNC Symbol;Acc:HGNC:438]

Gene symbols refer to the mouse orthologs corresponding to the deregulated proteins identified in the OGT-CDG mESC model. Disease associations are primarily based on human data from the DisGeNET CURATED database.

### Zscan4 Upregulation is Correlated With Increased Ogt-Tet1/2 Association

**Fig. 3:**
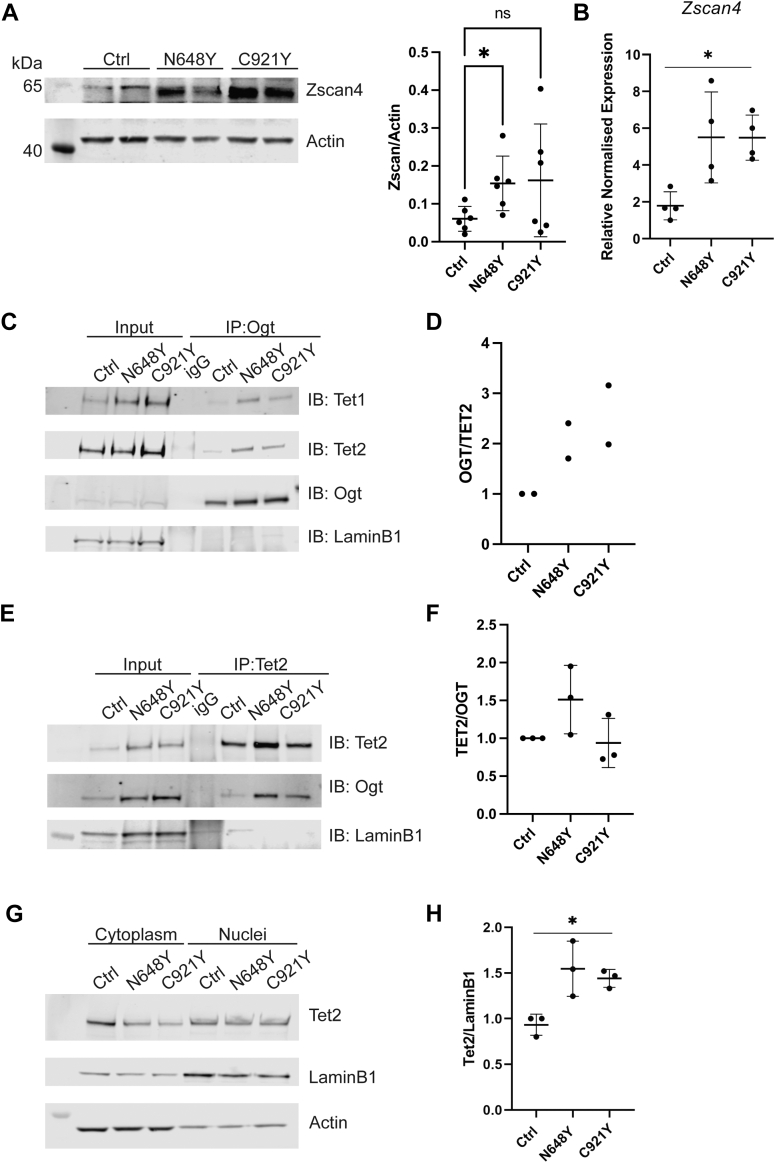
**Zscan4 upregulation and the Ogt-Tet1/2 complex.***A*, immunoblots showing Zscan4 and Actin levels in Control, N648Y, and C921Y undifferentiated mESCs and quantification of immunoblots of Zscan4 protein level in undifferentiated mESCs normalized to Actin signal. N = 6, mean ± SD. One-way ANOVA. *B*, qPCR analysis of gene expression of Zscan4 in undifferentiated mESCs. N = 4, mean ± SD. One-way ANOVA, ∗ corresponds to *p* < 0.05. *C*, Tet1 and Tet2 co-immunoprecipitated (CoIP) with endogenous Ogt from undifferentiated mESCs. Nuclear extracts were immunoprecipitated with anti-Ogt or rabbit IgG and probed with antibodies against the indicated proteins. Inputs loading controls are shown for all. LaminB1, a nuclear housekeeping protein, was included as a control to assess the quantity of nuclear material in the immunoprecipitates. *D*, quantification of the ratio of OGT to TET2 in OGT co-immunoprecipitated with endogenous TET2. n = 2. *E*, Ogt co-immunoprecipitated (CoIP) with endogenous Tet2 from undifferentiated mESCs. Nuclear extracts were immunoprecipitated with anti-Tet2 or rabbit IgG and probed with antibodies against the indicated proteins. Inputs loading controls are shown for all. *F*, Quantification of the ratio of TET2 to OGT in TET2 co-immunoprecipitated with endogenous OGT. n = 3, mean ± SD. *G*, Western blot analysis for Tet2, Actin (cytoplasmic marker) and laminin B1 (nuclear marker) in Control, N648Y and C921Y mESCs. *H*, Quantification of immunoblots on nuclear fraction of undifferentiated Control, N648Y and C921Y mESCs showing Tet2 levels normalized to Lamin B1 signal for nuclear fraction. n = 3, mean ± SD. One-way ANOVA. ∗ corresponds to *p* < 0.05.

Aberrations in Zscan4, which are implicated in facioscapulohumeral muscular dystrophy (FSHD) 1 and 2 - a disease characterized by progressive muscle weakness and atrophy (68) - arise in part from dysfunctions at the neuromuscular junction (NMJ) (69). We have previously shown that disruptions in O-GlcNAc cycling lead to significant deficits at the NMJ in *Drosophila*, resulting in impaired synaptic plasticity and motor function (70, 71, 72). Clinically, OGT-CDG manifests with hypotonia, a common feature of neuromuscular dysfunction that is consistent with the NMJ deficits observed in both FSHD and our *Drosophila* studies (51). The parallels between these conditions suggest that NMJ dysfunction may be a common outcome of perturbed molecular pathways that are sensitive to O-GlcNAc regulation, and we therefore further dissected Zscan4 as a mechanistic candidate for OGT-CDG. Zscan4 regulates genes such as KHDC1 and TRIM43, both of which are involved in FSHD (73, 74, 75). To confirm whether Zscan4 expression is affected in our model, we assessed its protein levels in OGT-CDG mESCs by western blotting as an orthogonal method to the mass spectrometry data (Fig. 3*A*). Although the changes were not statistically significant in the C921Y genotype, a trend toward upregulation of Zscan4 was observed in OGT-CDG mESCs compared to control cells (n = 6, mean ± SD, one-way ANOVA, ∗*p* < 0.05), perhaps reflecting the limited quantitative nature of this approach. However, qPCR analysis revealed a threefold increase in *Zscan4* mRNA levels in OGT-CDG mESCs compared to controls (Fig. 3*B*, n = 4, mean ± SD, one-way ANOVA, ∗*p* < 0.05). Together, these results suggest that OGT-CDG mutations lead to altered Zscan4 expression in this mESC model. Zscan4 expression is typically limited to the late 2-cell stage of mouse embryos and is essential for embryo implantation (76). In embryonic stem cell cultures, a small subpopulation exhibits 2-cell embryo-like characteristics, such as Zscan4 expression, increased histone mobility, and totipotency - i.e., the ability to contribute to both embryonic and extraembryonic tissues (77, 78). Zscan4 plays a crucial role in regulating pluripotency, genome stability, and telomere maintenance. Moreover, it can upregulate the expression of hundreds of genes during the late stages of induced pluripotent stem cell formation, leading to significant phenotypic changes (68). Several studies have demonstrated a correlation between Ten-11 translocation (TET) family proteins and Zscan4 expression (67, 79). Notably, a recent study has demonstrated a direct interaction between Zscan4 and Tet2, forming a transcriptional nexus that regulates metabolic rewiring and enhances proteostasis during reprogramming (79). This provides a potential mechanistic link between increased Zscan4 expression and altered Tet2 levels or activity in OGT-CDG mESCs. The interaction of Ogt with Tet proteins is critical for regulating CpG island methylation and transcriptional activation (20, 21, 80, 81). We therefore explored whether changes in the Ogt-Tet interaction could influence Zscan4 transcription. We performed immunoprecipitation of endogenous Ogt, revealing that the increased Ogt levels in OGT-CDG mESCs were associated with altered binding stoichiometry of Tet1 and Tet2 proteins (Fig. 3, *C* and *D*). Furthermore, immunoprecipitation of Tet2 resulted in increased co-immunoprecipitation of Ogt in OGT-CDG mESCs compared to controls (Fig. 3, *E* and *F*). This finding aligns with recent research on the Ogt-Tet2 interaction and its potential role in OGT-CDG syndrome (51, 82). Additionally, analysis of Tet2 protein levels in nuclear extracts demonstrated elevated Tet2 levels in OGT-CDG mESCs (Fig. 3, *G* and *H*, n = 3, mean ± SD, one-way ANOVA, ∗*p* < 0.05). These data suggest that Ogt upregulation leads to an increase in Tet2, enhancing the formation of the Ogt-Tet2 complex in OGT-CDG mESCs. In summary, our data suggest that OGT-CDG mutations result in elevated Zscan4 expression in mESCs, potentially through increased interactions between Ogt and Tet2 proteins. This mechanism may play a role in modulating chromatin structure and transcriptional programs critical for developmental processes, with possible implications for OGT-CDG pathology.

## Discussion

OGT-CDG is characterized by a spectrum of clinical features, with the most prominent being intellectual disability (ID) and neurodevelopmental delay (50, 52, 53, 54, 55, 56, 57), suggesting that the underlying molecular defects in OGT are critical for proper brain development and/or function. Protein O-GlcNAcylation, mediated by O-GlcNAc transferase (OGT), plays a pivotal role in various cellular processes, including transcription, signal transduction, and stress response. Disruptions in O-GlcNAc homeostasis have been linked to impaired neural differentiation and synaptic activity, both *in vitro* and *in vivo*, emphasizing the importance of O-GlcNAcylation in neuronal development and function (32, 46, 83, 84). Given that altered O-GlcNAc homeostasis in mESCs at the naïve stage may represent an early molecular contributor to the ID aspect observed in OGT-CDG patients, we employed TMT-based quantitative proteomics to investigate the downstream effects of OGT mutations. Our proteomics analysis identified several deregulated proteins that could provide insights into the molecular mechanisms underlying the neurological manifestations of OGT-CDG. Our results underscore a significant imbalance in the O-GlcNAc pathway in naive OGT-CDG mESCs. Western blot analysis revealed reduced global O-GlcNAcylation levels in OGT-CDG lines compared to controls, accompanied by consistent upregulation of Ogt at both the protein and mRNA levels (Fig. 1, *B*–*D*). While Oga levels showed minimal change at the protein level, mRNA levels were significantly reduced, further implicating a dysregulated O-GlcNAc cycle in the pathology of OGT-CDG. While this pattern of reduced global O-GlcNAcylation and Ogt upregulation has previously been observed for variants in the OGT catalytic domain (51, 52, 53, 54), this is not consistently so for TPR domain variants, suggesting possible distinct mechanisms by which catalytic and TPR mutations affect O-GlcNAc homeostasis and/or OGT-CDG (51).

Through TMT-based proteomics, we identified several genes linked to ID and microcephaly that emerged as candidates for further investigation (Table 1). For instance, *GDI1*, a gene associated with ID (score 0.80), has been implicated in synaptic vesicle trafficking and neuronal signaling, processes that are critical for proper brain function. Similarly, genes such as *PRKDC*, *ALPL*, and *KIF23*, which are linked to microcephaly (scores ranging from 0.40 to 0.45), are involved in key cellular functions such as DNA repair, motor protein activity, and cytokinesis. Although these proteins were not the primary focus of our study, their deregulation suggests that multiple cellular systems, ranging from genome maintenance and cytoskeletal dynamics to membrane trafficking, may converge to contribute to the neurological manifestations of OGT-CDG. These findings highlight the potential for broader proteomic dysregulation beyond the Zscan4-TET axis and support further investigation of these candidate pathways.

Among the deregulated proteins, Zscan4, a key marker in 2-cell stage embryos and 2-cell-like embryonic stem cells (ES), emerged as a candidate due to its role in maintaining genome stability and epigenetic reprogramming (76, 85, 86). Aberrations in *Zscan4* have been linked to Facioscapulohumeral Muscular Dystrophy (FSHD) (68) and we observed an increase in *Zscan4* mRNA levels in OGT-CDG mESCs. While the upregulation of Zscan4 was statistically significant at the protein level by Western blot in only one of the two OGT-CDG genotypes, both genotypes showed significant upregulation in proteomic and qPCR experiments. Recent studies proposing that TET proteins could be involved in the pathogenesis of OGT-CDG have drawn particular interest to Zscan and the TET family protein (51). TET proteins, which play a crucial role in DNA demethylation and chromatin remodeling, have been shown to regulate the transcription of Zscan4 (79, 87). The interaction between OGT and TET proteins is critical for TET-dependent chromatin remodeling and transcriptional regulation. OGT stabilizes TET1, enhancing the 5-hmC levels at TET1 target genes, while TET2 recruits OGT to chromatin substrates, promoting transcriptional activation (80, 81, 88, 89). Notably, recent work has also demonstrated that OGT can inhibit TET activity in heterochromatic regions, adding complexity to this regulatory network (90). Given the elevated Ogt levels observed in OGT-CDG mESCs, we hypothesized that these increases could sequester TET proteins, thereby altering their availability for other cellular functions. The upregulation of Ogt in our naïve OGT-CDG mESCs correlates with increased TET protein levels and the formation of the Ogt-Tet complex, perhaps contributing to dysregulation of transcriptional profiles. These findings are consistent with previous reports showing that OGT upregulation enhances TET1 and TET2 levels, influencing the broader transcriptional landscape (80, 89). While our data highlight important molecular candidates, including Zscan4 and TET, that may be involved in the OGT-CDG phenotype, these findings require further investigation. In particular, the role of the Ogt-Tet complex in transcriptional regulation, and its potential impact on genes critical for neurodevelopment, warrants additional study. To better understand the contributions of these proteins to OGT-CDG pathology, it will be essential to pursue further studies using cellular models involving neural differentiation that can more accurately capture the effects of OGT mutations in a physiological context.

In summary, our findings suggest that OGT-CDG mutations result in dysregulation of the Ogt-Tet complex, which in turn alters the transcriptional profiles of genes critical for development, including Zscan4 and several candidates linked to ID and microcephaly. This dysregulation may contribute to the neurodevelopmental and neuromuscular defects observed in OGT-CDG patients, providing new insights into the molecular mechanisms underlying this syndrome. However, further studies, including neural differentiation models and *in vivo* systems, will be crucial to fully delineate the role of OGT in these processes and to better understand the broader impact of OGT-CDG mutations.

## Data Availability

The mass spectrometry proteomics data generated in this study have been deposited to the ProteomeXchange Consortium via the PRIDE partner repository with the dataset identifier PXD064425 (access token: xYrFBA5sDqth) http://www.ebi.ac.uk/pride/archive/projects/PXD064425. All other data supporting the findings of this study, including processed proteomics outputs, R scripts for data analysis, are available from the corresponding author upon reasonable request.

## Supplemental Data

This article contains supplemental data.

## Conflict of Interest

The authors declare that they do not have any conflicts of interest with the content of this article.
