# Supplementary material for: Zscan4 as a Candidate Conveyor of Early Developmental Defects in O-GlcNAc Transferase Intellectual Disability
Source: Mol Cell Proteomics. 2025 Sep 29;24(11):101077. doi: 10.1016/j.mcpro.2025.101077 (PMC12744332; doi:10.1016/j.mcpro.2025.101077)
Supplement: Supplementary Tables [file mmc3.docx]

Supplementary figure legends

Supplementary Table 1: Primers used for RT-qPCR analysis of expression level of undifferentiated mESCs.

| List of Primers | Sequence (5’->3’) |
| --- | --- |
| *Actb* Forward | GATCAAGATCATTGCTCCTCCTG |
| *Actb* Reverse | CAGCTCAGTAACAGTCCGCC |
|  |  |
| *OGT* Forward | CCCCCTGAGCCCTTCAAAAC |
| *OGT* Reverse | TCGTTGGTTCTGTACTGTCGG |
|  |  |
| *OGA* Forward | TGCAGTGGTTAGGGTGTCG |
| *OGA* Reverse | AGCAAACGCTGGAACTCTCC |
|  |  |
| Zscan4 Forward | CAGATGCCAGTAGACACCAC |
| Zscan4 Reverse | GTAGATGTTCCTTGACTTGC |

Supplementary Table 2: List of Antibodies used for Western Blotting Analyses.

| Antigen | Producer and Catalogue Number | Dilution used |
| --- | --- | --- |
| OGT | SCBT (sc-74546) | 1:1000 |
| OGA | Sigma (HPA036141) | 1:500 |
| O-GlcNAc | Novus Biologicals (NB300-524) | 1:1000 |
| Actin | Proteintech (7D2C10) | 1:5000 |
| Zscan4 | Millipore (AB4340) | 1:5000 |
| TET1 | Active Motif (5D6) | 1:1000 |
| TET2 | CST (D6C7K) | 1:1000 |
| LaminB1 | Abcam (ab16048) | 1:5000 |
